# Surgical Repair of Cleft Lip: Comparison of Neonatal and Standard Timing in a Systematic Review

**DOI:** 10.7759/cureus.92858

**Published:** 2025-09-21

**Authors:** Kyle Walsh, Clare Foy

**Affiliations:** 1 Anatomy, Queen's University of Belfast, Belfast, GBR; 2 School of Medicine, Dentistry and Biomedical Sciences, Queen’s University of Belfast, Belfast, GBR

**Keywords:** cleft lip, neonatal management, paediatric surgery, plastic surgery, reconstructive surgery, surgical repair

## Abstract

Cleft lip and palate (CLP) abnormalities are common birth defects encompassing isolated cleft lip, cleft palate, or combined CLP. Current knowledge indicates that CLP has both genetic and environmental causes, with strong associations between a positive family history and maternal factors such as smoking, alcohol consumption, teratogenic substance use, and poor nutrition. The upper lip develops as a result of the fusion of the paired medial nasal prominences to the maxillary prominences, forming the philtrum and lateral portions of the upper lip, respectively. Cleft lip, therefore, arises from a failure in those named structures to fuse. The current best treatment involves surgical repair to reconstruct the lip to restore normal appearance and function, including feeding and speech. This normally occurs around the age of six months (standard time) in most centers, but is performed much earlier, in the neonatal period, in other centers. This study aims to determine whether neonatal cleft lip repair is superior to standard time repair. Secondary aims are to determine both the feasibility and the safety of neonatal repair. Advanced literature searches were carried out using Medline ALL (1946 to date) and Embase (1974 to date); 11 articles were deemed relevant and included in this study. Aesthetic results showed excellent outcomes with neonatal repair with regard to the appearance of the scar, facial (lip and nasal) symmetry, but those aesthetic results are no better than those achieved at standard time. Although early intervention can be beneficial as early repair takes place when the cleft is less severe and when the tissues are more malleable, making the surgery less challenging, and when some aspects of fetal scar healing remain. Additionally, early repair has a positive impact on the development of the alveolar projections and can assist in reducing an alveolar cleft if present, improving the aesthetic outcome. Moreover, neonatal surgery carries with it no greater risk than surgery carried out at six months and will allow feeding to begin at an early stage, promoting recovery. Early repair also brings with it a large positive psychosocial impact, where infants and mothers can build a normal relationship from an early stage. Later in life, children and adults will be less self-conscious following good aesthetic repair. In conclusion, based on the limited available evidence, neonatal repair may be recommended over standard time repair.

## Introduction and background

Cleft lip and palate (CLP) abnormalities are the most common congenital orofacial anomalies. More commonly, they occur as isolated deformities but can be associated with many other clinical conditions, in particular congenital heart disease, but also over 300 different documented syndromes can display CLP deformity. The most common is the Pierre Robin sequence, which includes isolated cleft palate, retrognathia, and glossoptosis. Other syndromes of note include Stickler, Shprintzen, and Down syndrome. Therefore, pediatric assessment must be aimed at identifying an underlying syndrome if CLP is present [1]. Surgical intervention is almost always required for aesthetic outcomes as well as to improve functional outcomes in terms of feeding. This paper will review the incidence and etiology of CLP as well as the development and surgical repair of CLP in the following sections. The aim is to determine whether neonatal repair of CLP is superior to standard-timing repair. Secondary aims are to assess the feasibility and safety of early repair.

Incidence

According to the Cleft Lip and Palate Association (CLAPA), the incidence of CLP in the United Kingdom is about 1 in 700 newborns, equating to approximately 1,000 new cases each year [2]. More specifically, there were 27 new cases in Northern Ireland [3] out of a total of 22,833 live births, giving a prevalence of 0.12% [4]. Incidence varies between ethnic groups; it is more common in the eastern Asian population and less common in the Black population.

Many subgroups of CLP exist. These include, with typical distribution, isolated cleft lip (24%), isolated cleft palate (45%), and combined CLP (31%). The combined CLP subgroup can be further divided into unilateral (22%) and bilateral (9%) [1].

Males are more commonly affected with a combined CLP, whereas females are predominantly affected by an isolated cleft palate [5]. In those cases of isolated unilateral cleft lip, the left side is more commonly involved (60%) [1]. Cleft lip with or without cleft palate and isolated cleft palate are those subtypes that are most commonly associated with other congenital abnormalities. Those individuals with isolated cleft palate display the greatest frequency of additional congenital anomalies. In a European epidemiological study involving 4,000 individuals, isolated CLP deformities made up 55% of all cases, 18% were associated with other congenital anomalies, and the remaining 27% of cases were diagnosed as being caused by recognized syndromes [6].

Etiology

Current opinion suggests that the etiology of CLP is based on both a genetic predisposition and contributing environmental factors [7]. Both genetic and environmental factors play a role in syndromic causes of CLP, but the etiology of isolated CLP is poorly understood [8]. In terms of genetic factors, a family history of CLP in which a first-degree relative is affected increases an individual’s risk to 1 in 25 live births [9]. Case studies have revealed that both chromosomal abnormalities and single gene mutations are associated with the development of CLP [8]. A strong genetic component has been found in monozygotic twins with CLP, and there are also environmental factors at play, reinforced by the fact that there is a higher risk between dizygotic twins compared to singleton siblings [10]. Many important environmental risk factors have been linked to CLP, including maternal exposure to alcohol, tobacco smoke, poor nutrition, viral infection, and teratogenic substances, including medications [9]. There is a population-attributable risk of up to 20% for those mothers who smoke during pregnancy; this may be a confounding factor in maternal alcohol use [11]. Poor nutrition has also been linked to the development of CLP. Meta-analysis of multivitamin use during pregnancy is thought to be associated with a 25% reduction in CLP prevalence, showing the importance of zinc and vitamin B6, as deficiencies in both have been linked to increased prevalence of CLP [12,13]. Folate deficiency and folate antagonists have been associated with an increased risk of CLP [14], though many case-control studies remain inconsistent [9]. The most important teratogenic medications associated with CLP are the anticonvulsant drugs diazepam, phenytoin, and phenobarbital, as well as maternal corticosteroid use during pregnancy [15,16]. In short, genetic influence plays a greater role in CLP compared to environmental factors, which can be seen to be influential in the cause of isolated cleft palate; these factors disrupt normal development at a critical stage of development [17].

Normal facial development

Development of structures of the head and neck arises from the pharyngeal arches (PA), which appear in the fourth and fifth weeks of embryological development. They resemble bars of mesenchymal tissue separated by pharyngeal clefts. The PA will, in time, form the structures of the face and neck. By the end of the fourth week, the stomodeum or primitive mouth, surrounded by the first pair of PA, will be present in the center of the embryo’s face. By day 42 of development, five mesenchymal prominences are present: paired mandibular and maxillary prominences and the frontonasal prominence, which have arisen from neural crest cells differentiated into ectomesenchyme that migrates over the face [18].

Development of the upper lip

The formation of the upper lip begins at day 24 and is completed by day 37. In the fifth week, nasal placodes, thickenings of ectoderm on the frontonasal prominence, invaginate to form nasal pits. This divides the frontonasal prominence into paired medial and lateral nasal processes. During week 6, the medial nasal processes will move medially and fuse as the maxillary prominences increase in size. The intermaxillary segment (process) is formed from the fusion of the paired medial nasal prominences both superficially and deep, and is composed of three components: a labial component, which will form the philtrum of the upper lip, as well as jaw and palatal components. The upper lip will therefore be formed from the paired medial nasal prominences, which form the philtrum of the lip, and the maxillary prominences, which form the lateral portion of the upper lip. In contrast, the lower lip is formed from the fused mandibular prominence [19].

Clefting of the upper lip can arise both unilaterally and bilaterally. A unilateral cleft lip results from a failure in fusion of the maxillary prominence to the labial component of the intermaxillary segment on the affected side, more commonly the left side [20]. In around 20% of cases, a Simonart’s band is present. This is a band of lip tissue that bridges the cleft lip [21]. Complete cleft lips extend into the nose, whereas incomplete ones do not.

The abnormal appearance is a consequence of disruption of the surrounding rings of facial muscles, known as the rings of Delaire. In this scenario, the nasolabial and bilabial muscle rings are disrupted, giving the characteristic asymmetrical deformity involving displacement of nasal skin onto the upper lip as well as retraction of the upper lip, resulting in distortion of the vermilion border [22]. Additionally, in complete cleft lips, a characteristic nasal deformity can be seen in which the caudal septum and columella are pulled to the non-cleft side through the unopposed action of orbicularis oris [23]. The bony nasal septum is deviated to the nostril on the cleft side, and on the cleft side, there is a lack of bony support for the alar cartilage, causing inferior, lateral, and posterior displacement. Overall, this forms a wide, horizontal nostril with a flattened alar dome [24]. Bilateral cleft lip occurs when the maxillary prominences fail to unite with the fused medial nasal prominences bilaterally. This defect is particularly deforming because the orbicularis oris muscle is discontinuous around the mouth, resulting in nostril flaring and the formation of a prolabium, lip tissue devoid of muscle, which projects inferiorly and anteriorly [20].

Surgical management

Surgical repair is the definitive treatment of CLP deformities, but treatment of CLP is multidisciplinary, requiring input from craniofacial surgeons, otolaryngologists, and geneticists [25]. The goals of surgery are twofold: the first being reconstruction of the CLP. Achieving normal or near-normal anatomy allows normal function of the lip and palate and, consequently, normal development. This involves rebuilding a complete and competent oral fissure in which orbicularis oris surrounds the fissure, allowing for normal speech, feeding, and emotional facial expression [22]. Additionally, simultaneous repair of the palate is based on restoring normal anatomic function, preventing nasal reflux of food and secretions, and allowing normal mastication and swallow to occur. The second aim is cosmetic reconstruction of the lip. This aesthetic result can be challenging and can carry a huge psychosocial burden. Cosmetic reconstruction is based on many principles, including the formation of a correctly aligned and symmetrical cupid’s bow, vermilion border, and philtral column [26], as well as limiting the size and strategic positioning of the scar and creation and positioning of the nasal floor, nasal sill, and alar cartilages [27].

Surgical techniques

The specific surgical techniques and procedures used depend on the defect present. Cheiloplasty is the term used to describe surgical lip restoration [28]. The following techniques described only repair the defective cleft lip. The Millard technique [29] is a rotation advancement repair, used most commonly in the United States, for the repair of a unilateral cleft lip [30]. This method is popular for many reasons, including its flexibility and applicability; it is referred to as the *cut-as-you-go* method [31]. This technique allows continuous modifications during the procedure and produces a minimal scar concealed along the philtral border [30]. Furthermore, it allows access to the nasal cartilages for simultaneous rhinoplasty in which early repair can lead to a better cosmetic outcome with a more symmetrical nasal appearance and a reduction in secondary surgeries [26]. Although this generally successful technique is challenging and relies on surgical experience and artistry [32], it may result in whistle deformity due to wound contracture and in nasal vestibular stenosis [33]. Many refinements have been made to Millard’s original technique [29], giving rise to the Millard II technique. Further modifications have since been proposed [34]. Tennison [35] described a triangular flap technique, revised by Randall [36], for the repair of a unilateral cleft lip, which is similar to the Millard technique [29]. This geometric method is based on precise measurements and, therefore, prevents error and, therefore, is more suited to less experienced surgeons. The triangular flap forms a zigzag scar, which is unfortunately more noticeable but less prone to contracture compared to the Millard technique [37]. In the setting of a bilateral cleft lip, the straight-line technique is the recognized universal method in which the prolabium is used to reconstruct the philtrum [38].

Timing of surgery

The timing of surgery was first addressed by Wilhelmsen and Musgrave, who in 1966 described the rule of 10s for safe neonatal surgery. A child is fit for surgery when they weigh more than 10 lbs, have a hemoglobin over 10 g/dL, and a leukocyte count of less than 10,000 cells/mL [39]. Current standard practice is to repair the cleft lip during infancy, typically between 6 and 12 months of age, while palate repair is deferred until around 18 months [40]. Although there is no general international consensus, standard practice involves a single surgical repair of isolated cleft lips around the age of six months [1]. More specifically, unilateral cleft lip repair is typically performed at five to six months of age, whereas bilateral cleft lip repair is carried out slightly earlier, at four to five months [1]. Where CLP occur together, repair involves two separate operations. The first addresses the soft tissues, the lip and soft palate, followed by reconstruction of the hard palate at a later date [1]. It is widely accepted that early closure of the palate improves speech, whereas later closure favors maxillofacial growth [41]; therefore, a balance is made and repair of the palate occurs.

Although these timings represent standard practice, not all centers adhere to them, and some perform successful cleft lip repairs in the neonatal period. As of 2005, 33.3% of centers worldwide were undertaking primary cleft lip repair before the child was one month of age. The remaining 65.9% and 0.7% of centers performed repairs between three and six months of age and after six months of age, respectively [42]. Debate therefore persists regarding the optimal timing of intervention, balancing surgical outcomes against anesthetic risks. This study aims to compare the advantages and disadvantages of neonatal cleft lip repair with those of standard-timing cleft lip repair.

Methodology

Medline ALL (1,946 to date) and Embase (1974 to date) databases were used to carry out an advanced search for relevant literature using the following search terms: “cleft lip” AND “surgery” OR “repair” OR “plastic surgery” OR “reconstructive surgical procedures” AND “neonatal,” as seen in the search strategy given in Table 1.

**Table 1:** Search strategy. / is a topic search; .mp. is where the terms are searched using keywords.

Search strategy (Search term and number of results)	
1	cleft lip/ (27326)
2	surgery/ (537834)
3	repair.mp. (731653)
4	plastic surgery/ (84393)
5	Reconstructive Surgical Procedures.mp. or reconstructive surgery/ (55415)
6	2 or 3 or 4 or 5 (1356548)
7	1 and 6 (5337)
8	neonatal.mp. (477047)
9	7 and 8 (105)

The final 105 relevant articles were screened on the basis of their title and abstract. Screening was performed by two independent reviewers. Titles and abstracts were reviewed, followed by full-text assessment. Disagreements were resolved by discussion. Inclusion criteria were defined using the PICO framework:

P: neonates with unilateral or bilateral cleft lip (with or without cleft palate) I: surgical reconstruction/repair of the cleft lip in the neonatal period C: comparison with standard-timing repair or evaluation of neonatal repair alone O: any aesthetic/cosmetic surgical outcome

**Figure 1:**
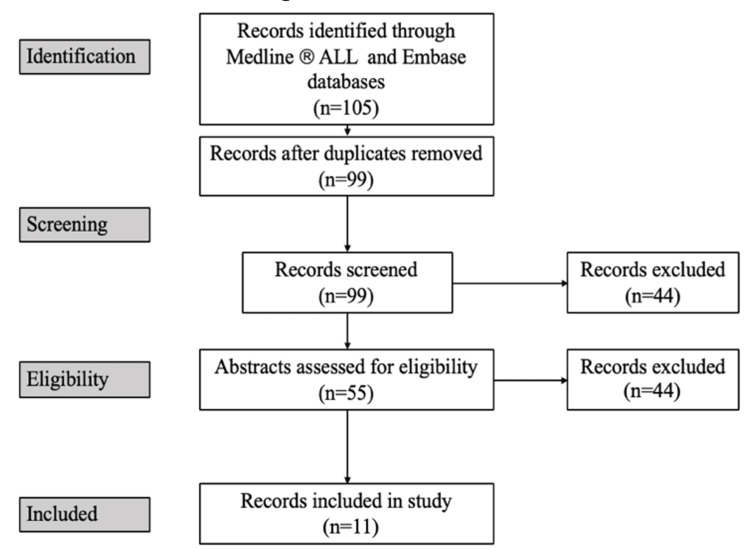
PRISMA flow diagram depicting the article selection process. PRISMA, Preferred Reporting Items for Systematic Reviews and Meta-Analyses

Any articles related solely to developmental or growth outcomes were excluded. Articles were also excluded based on relevance, for example, those related to irrelevant syndromes, not including neonates, molecular or cellular research, or surgical outcomes related to complications or anesthetic safety only. No articles were excluded based on date. In total, 11 articles were deemed relevant based on their aim to evaluate or compare neonatal cleft lip surgery and are therefore included in this study, along with a summary of the included studies (Figure 1, Table 2).

**Table 2:** Summary of included studies. CLP, cleft lip and palate

Author	Study title	Year	Aim	Number of participants and methods	Findings
Borsky et al. [43]	Our First Experience with Primary Lip Repair in Newborns with Cleft Lip and Palate	2007	To present the results of the first newborns to undergo CLP repair surgery in the first week of life.	44 newborns with CLP are repaired using the modified Tennison method.	Results show excellent aesthetic outcomes regarding lip scarring and nose appearance. There is no impact on maxillary development.
Jiri et al. [44]	Successful Early Neonatal Repair of Cleft Lip Within First 8 Days of Life	2012	To assess the surgical outcomes of performing cheiloplasty in early newborns with CLP.	97 neonates with CLP were repaired using the modified Tennison technique. The aesthetic outcome was measured according to scar visibility and the symmetry of the lip and nose shape.	Early neonatal CLP repair shows good aesthetic results and has an important psychological impact on the child and family.
Calteux et al. [45]	Neonatal Cleft Lip Repair: Perioperative Safety and Surgical Outcomes	2013	A retrospective study into the perioperative safety and surgical outcomes of neonatal cleft lip repair.	42 neonates with unilateral or bilateral cleft lip operated before the 26^th^ day of life using the triangular skin flap technique (Randall-Tennison).	Neonatal cleft surgery does not pose any problems related to anaesthesia. Independent reviewers rated the scars as excellent.
Cerny et al. [46]	Our Experience with Lip-Nose Cleft Repaired in the Neonatal Period	2010	A report on the experience of neonatal lip-nose cleft repair.	161 newborns underwent repair of unilateral and bilateral cleft lip using the modified Tennison technique at a median age of 5 days old.	Early cleft lip repair provided a superb aesthetic outcome as rated by the plastic surgeon and parents. There were no negative effects on cranial morphology.
Freedlander et al. [47]	Neonatal Cleft Lip Repair in Ayrshire; a Contribution to the Debate	1990	A report on the results of 10 years of neonatal cleft lip repairs.	31 neonates underwent cleft lip repair for unilateral or bilateral cleft lip and or palate within the first 48 hours of life using the Millard method for all but 2 repairs (Tennison and Manchester repairs).	According to a grading system used to assess aesthetic outcome, 11 cases were rated as excellent/good, 9 satisfactory, and 6 poor. 27% of cases underwent secondary surgery for lip revision. Neonatal surgery gives good results and can offer advantages to the parents and child.
Goodacre et al. [48]	Does Repairing a Cleft Lip Neonatally Have Any Effect on the Longer-Term Attractiveness of the Repair?	2004	To determine whether the attractiveness and success of surgical outcome differ depending on the time of repair.	A blind and randomized trial in which surgeons and lay panellists reviewed photographs and videos of neonatal cleft lip repair (n = 50, median age 4 days old) compared to late repair (n = 60, median age 104 days old). Compared to a normal control group (n = 100).	There was no comparable difference between early and late repair, as both groups were rated similarly successful and attractive; video analysis favours early repair.
Hammoudeh et al. [49]	Early Cleft Lip Repair Revisited: A Safe and Effective Approach Utilizing a Multidisciplinary Protocol	2017	To design a protocol for safe and effective early cleft lip and nasal repair to mitigate anaesthetic and surgical complications by comparing early and late repair of CLP.	32 patients underwent CLP repair at a mean age of 34.8 days (13-69 days). 3D images were compared pre-and post- operatively for analysis and comparison.	Early repair improves nasal symmetry, which statistically improves the symmetry of anatomical landmarks.
Lazarou [50]	Comparison of Neonatal Cleft Lip Repair to Standard Time Repair Done by the Same Surgeon	2016	A retrospective comparison of the advantages and disadvantages of neonatal cleft lip repair compared to standard time repair (3 months) by the same surgeon.	127 neonatal repairs (between day 1-8) compared to 136 standard time repairs (3 months of age).	Neonatal repair is preferred based on the surgeon’s opinion on outcome (aesthetics and complications) and parent satisfaction (aesthetics, feeding).
Le Pendeven et al. [51]	Long-Term Morphologic Results of a 32 Successive Patients Series Presenting Unilateral Complete Cleft Lip and Palate with Surgery at Early Age	2009	To describe and evaluate the long-term morphological results of patients undergoing neonatal repair of unilateral cleft lip.	32 patients with unilateral CLP underwent surgical repair using the Millard technique at an average age of 70.4 days old.	Neonatal cleft repair was satisfactory, although 87% of patients required secondary operations for minimal lip corrections, palate changes, and rhinoplasty.
McHeik et al. [52]	Early Repair for Infants with Cleft Lip and Nose	2006	To study the aesthetic results and dental arch relationships 10 years after neonatal cleft lip repair.	123 patients underwent cleft lip and nose repair using the Millard technique at an average of 13.5 days old. 40 of these patients were operated on in the first week.	Patients displayed excellent aesthetic quality in the lip and nose repair with symmetrical anatomical landmarks. 28% of patients required minor surgical revision.
Valentova Strenacikova et al. [53]	Primary Repair of Cleft Lip and Nose in the Neonatal Period	2018	To compare the surgical outcomes of patients repaired for CLP in the neonatal period with those at 3 months.	A total of 571 cases are included in this trial, of which 83% are repaired early (between 0 and 3 weeks) or late (after week 3 and usually before 3 months)	Early repair is advantageous as it results in an almost negligible scar, tissues are more formable, and there is a huge family psychosocial benefit. Patients feed and gain weight as non-cleft children would.

In this systematic review, 1,181 neonates were identified who underwent early or neonatal repair, that is, within 30 days of birth, and 325 babies who underwent standard time repair, that is, beyond 30 days but typically between three and six months of age.

## Review

Results

Aesthetic Outcomes

In terms of the aesthetic outcomes of neonatal surgery, it is important to consider the appearance of the scar, facial (lip and nasal) symmetry, as well as the need for secondary revision surgery. From data published by Calteux et al. [45], all 42 neonates were reviewed by independent adjudicators who rated the scars as excellent as per the Vancouver Scar Scale. None of these primary repairs required further revision at a later date. Although in the same study, they reported slight labial asymmetry between the two halves of the lip. Likewise, results from Cerny et al. [46] showed a *superb aesthetic outcome* of the scar as rated by both plastic surgeons and parents; similar results were reported by Borsky et al. [43] and McHeik et al. [52]. Jiri et al. [44] also reported *very good aesthetic* results. In the early repair of cleft lip in 97 neonates, a lip scar was barely visible in 83.5% of patients (1% clearly visible), with symmetry achieved in 64.9% of noses and 68% of lips, 8-12 months after cheiloplasty.

In a robust study by Goodacre et al. [48], both surgical specialists and lay people reviewed postoperative photographs and videos in a randomized, blind fashion comparing early and late repair. Results from the photographs showed no difference in aesthetic outcome as they were reported *similarly attractive*, but when videos were reviewed, results were significantly different, in favor of early repair. In contrast, Le Pendeven et al. [51] reported that, in their study of 32 consecutive patients, their aesthetic outcomes were *satisfactory*. However, there were postoperative abnormalities in 53% (red lip discontinuity and mucous excess), and 87% of patients required, albeit minor, revision surgery.

Surgical Techniques and Revision Rates

In particular, more successful results were observed when the palate and lip were reconstructed separately compared to simultaneous lip and palate repair. Lower revision rates have been published by Freedlander et al. [47]. In their study of 31 patients, 84% underwent cleft lip repair within the first 48 hours of life. The 31 patients were rated for aesthetic outcome: 11 were rated as excellent, 9 as satisfactory, and 6 as poor. The remaining patients were not followed up for various reasons. Seven (27%) of those reviewed required revision surgery for lip scarring or vermillion symmetry. Similarly, Mcheik et al. [52] reported a revision rate of 28% in their study (35 out of 123 cleft lip repairs) for minor adjustments to the lip borders and nasal cartilages.

Scarring and Facial Symmetry

In addition to scarring, observations have also been made on facial symmetry. Mcheik et al. [52] reported “excellent aesthetic quality” in terms of lip and nasal symmetry, including a symmetrical cupid’s bow, philtral columns, nostrils, and nasal sills. Further improvements in nasal symmetry were reported by Hammoudeh et al. [49], who performed early cleft lip repair (between 13 and 69 days of age) using a modified subunit repair technique. Statistically significant improvements were seen across four different anthropometric measurements using anatomical landmarks such as columella angle and length ratios, as well as nasal base width and nostril height ratios.

Psychosocial and Parental Satisfaction

The outcome is the psychosocial aspects of early repair. Results from many of the studies highlight the parents’ satisfaction with early repair. Lazarou et al. [50] reported the reduction in “emotional and psychological strain” based on the ability to breastfeed and reduced hospital stay. Further to this, Jiri et al. [44] explained how parents prefer to bring home and present a newborn to family and friends without a malformation present.

Discussion

Aesthetics

Performing reconstructive cleft lip repair in the neonatal period may be favored for several reasons. The studies on the whole report a good aesthetic outcome when compared to standard time repair, describing the ability to achieve *excellent* scars as well as satisfactory facial symmetry. A possible explanation for this is that neonatal tissues are more formable, making the surgical repair less challenging [53]. Hammoudeh et al. [49] also described the benefits of early intervention when the nasal cartilages are most moldable, facilitating repair and resulting in a long-lasting symmetrical outcome. Bromley et al. [54] noted the benefit of neonatal lip closure when the alveolar segments are most malleable. Furthermore, Nakajima et al. [55] described how the nasal skin and cartilage are softer and more malleable in the neonate compared to those one month older. Hammoudeh et al. [49] explained how earlier intervention allows release of muscular attachments from the nasal cartilages, theoretically arresting the progressive lip and nasal deformity, allowing for a less severe cleft and a less challenging repair in the neonatal period compared to that at three to six months. Those in favor of standard-time repair report better aesthetic results when the child is larger and the anatomical structures are more developed [56], which contrasts with the aesthetic outcomes observed with early intervention. Stark [57] observed that the lip grows less than 2 mm vertically in the first three months of life, casting doubt on the claim that delaying repair makes the operation less challenging because the structures are larger. Although the aesthetic results are successful, there is little evidence to show that outcomes in the neonatal period are superior to those achieved with standard-time repair.

The advent of fetoscopic surgery has provided insight into the benefit of early intervention for many conditions, including CLP [58]. Fetal wound healing has been investigated through both animal and human models as far back as the late 1970s and has shown that fetal wounds can repair rapidly in the absence of scar formation [59]. In response to injury, fetal dermal tissue can regenerate a “non-disrupted collagen matrix identical to that of normal tissue,” preventing scar formation [60]. Le Pendeven et al. [51] explained that newborns may retain the ability for fetal-like tissue repair, either with or without scar formation. In-utero and endoscopic repair of cleft lip and alveolus in lambs at day 75 of gestation has resulted in healing without scar formation [61]. This, therefore, represents an extremely attractive area of research for plastic surgeons, as it offers the potential to repair a cleft lip scarlessly before birth. Repair of human fetal CLP has been reported only once in the literature, by Ortiz Monasterio [62], in which the infant died two months after discharge. Currently, the feto-maternal risks outweigh the benefits of repairing CLP, and fetal surgery therefore remains unacceptable for non-life-threatening conditions [63].

It is thought that, although human fetal scarring begins at approximately 24 weeks of gestation [64], earlier intervention, such as in the neonatal period, results in better scarring outcomes [49]. In addition to scar appearance, Jiri et al. [44] reported that patients undergoing early repair experience faster scar maturation, four to five days compared to seven to eight days for those operated on at three months.

Surgical Technique

When repairing the cleft lip in the neonatal period, it is important to understand which surgical techniques are most favorable. In the studies included, most centers performed cheiloplasty using the Tennison or Randall-Tennison technique with successful results. One exception is the study by Le Pendeven et al. [51], who performed unilateral CLP repairs using the Millard technique and reported satisfactory results. The more striking statistic is that, in this study alone, 87% of patients required secondary revision surgery at a later date. This may be explained by the technique used, as other studies have reported the benefits of the Tennison technique in the neonatal period. One such study, by Nakajima et al. [55], reported that very few patients developed a nasal deformity, and therefore fewer required revision surgery. Moreover, compared to the Millard technique, the Tennison technique produced a straight-line scar when repairing bilateral cleft lips, which is fine in nature and can be placed along the philtral column, resulting in a more symmetrical cupid’s bow. In the 117 cases repaired with early surgery, the scars resulting from the Tennison technique were far more acceptable than those produced by the Millard technique.

Facial Growth and Development

The timing of cheiloplasty has an effect on the growth of the surrounding facial skeleton and structures [65] as well as the development of the jaw segments. Repair of the lip resumes the continuity of the orbicularis oris muscle, which has a significant impact on the development of the alveolar projections [65]. This consequently has a great effect on the facial appearance of an individual in the first year of life and can vastly improve the aesthetic outcome [44]. Positive effects of early surgical intervention by Valentova Strenacikova et al. [53] have shown a significant improvement in the aesthetics of the middle third of the face. Akin et al. [66] described the effect of early cheiloplasty on the alveolar cleft, showing that early closure of the lip had an "occlusive effect" on the alveolar arch. It is understood that when repaired, the lip acts as a sphincter and the tongue functions as an obturator, closing the opening in the alveolus and thereby molding the dental arch. This mechanism is more effective than using orthodontic devices to achieve extraoral traction [66].

Further work by Huang et al. 2002 [67] supports this phenomenon. In their study, dental casts were used to evaluate maxillary dental arch development in infants with unilateral complete CLP, providing a three-dimensional assessment of the effect early cheiloplasty has on the growth and development of the clefted jaw segment. They concluded that a repaired lip could exert continuous pressure on the anterior aspect of the dental arch and therefore would decrease the width of the anterior maxillary cleft as it grew. Moreover, the pressure exerted would mold the maxillary arch through a *bone-bending* effect. In line with this, Jiri et al. [44] published data using 3D geometric morphometric methods. In short, data at three months of age showed growth of the segments toward each other when cheiloplasty was carried out within the first eight days of life.

Anesthetic Complications

The key question for any surgical procedure is whether the benefits outweigh the risks. As CLP abnormalities are non-life-threatening, repair in the neonatal period must be absolutely safe to warrant its use. As previously described, Wilhelmsen [39] reported on the complication rates in CLP operations, demonstrating a fivefold increase if the child did not follow the rule of 10s. For many years, plastic surgeons were, therefore, reluctant to operate in the neonatal period, the first 28 days after birth [68]. Now, 53 years on, our understanding and techniques in neonatal anesthesia and surgery have improved drastically, with more recent studies indicating the contrary [56]. Still, the risk of general anesthesia in neonates remains the key factor for centers opting for standard-time repair [56], with a reported rate of perianesthesia cardiac arrest of 24 per 1,000 procedures [69].

Many advocates of neonatal surgery have been carrying out cleft lip repair with strict exclusion criteria, including low birth weight, respiratory issues, hypoglycemia, jaundice, and congenital heart defects [47,52], in order to reduce anesthetic complication rates. Oral clefts may be syndromic and present with other congenital defects such as heart defects [47]. Congenital heart abnormalities remain a key contraindication for neonatal repair, with cardiac abnormalities present in 5.9% of those with oral clefts [70]. The significance of this is that cardiac defects double the risk of perioperative mortality [71]. Surgery at such a young age may not allow sufficient time for a medical or congenital abnormality, such as this, to be diagnosed [47].

A study by Harris et al. [72] investigated the complication rates in the repair of cleft lip in all children, with no strict exclusion criteria, except those with unacceptable anesthetic morbidity. Ninerty-nine consecutive repairs were performed within the first 28 days of life with an average birth weight of 3,300 g (7.28 lbs). Complication rates were low, with one case of hypoxia and five cases of nasal obstruction, with all patients recovering without any long-term effects. Harris et al. [72] concluded that there was no evidence of neonatal repair being unsafe. Further work by Galinier et al. [56] showed similar results in a retrospective study involving 61 neonatal children undergoing repairs for cleft, alveolus, or palate. Operations took place at an average age of 7.5 days, with a mean weight of 3,190 g (7.03 lbs) and an American Society of Anesthesiologists (ASA) physical status classification of 1-2. All children underwent a preoperative malformation workup to screen for underlying congenital abnormalities. This tool could be used or adapted by any centers undertaking preoperative screening before cleft lip surgery. There were no surgical or major anesthetic complications; four neonates had minor intubation difficulties, and one neonate experienced an episode of desaturation (SpO₂ <90%) with bradycardia. This episode occurred in a child with known underlying cardiopathy, including ventricular and atrial septal defects and pulmonary arterial hypertension. However, opposing evidence in the literature indicates an increased risk in the neonatal period [73]. This study reported a higher risk of both cardiac arrest and death during operations performed within the first 72 hours of life. It should be taken into consideration that 67% of those patients enrolled in this study underwent major cardiac, vascular, thoracic, or abdominal surgery with an ASA score of 3-5. More recent data suggesting an increased risk in neonatal surgery indicated that these risks could be attributed to surgical problems or changes in the child’s condition prior to surgery [74]. The safety of anesthesia was recently questioned following data from numerous animal studies showing that exposure during this critical period of neurodevelopment could lead to neurodegeneration and abnormal synaptic development, with subsequent deficits in functioning and learning later in life [75]. However, emerging robust studies using human data do not support these findings, suggesting that other factors may be at play [76].

Based on the current evidence, it is fair to conclude that neonatal cleft lip repair is safe, with no evidence to suggest otherwise [72]. Stark [57] recommended that cleft lip repair be carried out as early as possible and that the first two weeks are the safest time. Cannon [77] further stated that the operation can be performed within the first 24 hours of life, provided the neonate is healthy, and that with proper anesthesia and supportive care, the risk of surgery is no greater than when performed several weeks later. Furthermore, a review of the literature on cleft lip repair in the neonatal period shows similar complication rates for procedures performed neonatally compared with those performed at the standard time [56].

Feeding

Nutrition is of utmost importance for any child. The presence of a CLP can pose challenges for feeding in both mothers and their newborns before repair [78]. The oronasal communication in CLP, or the discontinuity of the lip in a cleft lip, impairs the ability to generate the negative pressure required for sucking [79]. Children awaiting surgery may benefit from the use of a feeding appliance or a modified bottle [80]. Early repair can address feeding difficulties, allowing the child to feed as normally as possible from an early age. Desai [81] reported on his experience with early CLP repair within the first 16 weeks of life, noting that a clear indication for early surgery is a child who is failing to thrive. Children who are failing to gain weight may warrant postponement of surgery, but repair of the defect allows normal feeding to begin. After the operation, the children gain weight quickly, and the mothers report satisfaction.

Furthermore, a publication by Mzezewa et al. [82] demonstrates the benefits of early intervention in infants struggling to breastfeed. In this report, 23 neonates underwent CLP repair at a median age of nine days, with no mortality and minimal morbidity. Postoperatively, all successfully breastfed and showed weight gain above the 50th percentile on health charts. Immediate, unrestricted postoperative feeding, such as breastfeeding, has no detrimental effects on the outcome of surgical repair and is beneficial from nutritional, immunological, and psychological perspectives [83]. Adequate postoperative nutrition supports optimal wound healing [84] by promoting an anabolic growth phase in the newborn [47]. Neonatal intervention has, therefore, proven advantageous compared with standard-time repair in cases of problematic feeding.

Psychological Impact

Early repair of the cleft lip can have a great psychological impact in many ways [85,86]. Tobiasen [87,88] demonstrated a strong correlation between the attractiveness of the repair and later psychological effects. More specifically, patients with repaired cleft lips are at a significantly higher risk of social competence challenges, including difficulties in developing friendships, progressing in school, and participating in organizations or groups. Interestingly, these patients are not at higher risk of developing psychopathology compared to individuals without a cleft [89]. Therefore, early successful repair, resulting in excellent aesthetic outcomes later in life, can help offset negative psychological impacts, such as self-consciousness. The mother’s psychological status has also been investigated by Natsume et al. [90], who revealed that mothers are more seriously affected by a visible cleft compared to an invisible cleft, e.g., cleft palate only, with some mothers even considering suicide. It makes sense that earlier intervention can have psychosocial benefits compared to standard-time repair. A fair argument against early repair is that separating the mother and child to perform the cleft repair during a critical bonding period can be detrimental. However, early repair allows the family to overcome the deformity sooner, enabling them to focus on healing as well as normal aspects of their child’s development, such as feeding and bonding [49]. Early repair facilitates normal socialization from as early as the first few days of life, improving the mother-infant relationship, and thus approximates the scenario of a child without a cleft, where the parents can bring home and present their baby to family and friends without a cleft [44].​​​​​​​

Diagnosis

Diagnosis of CLP can be made using prenatal ultrasonography during the second and third trimesters of pregnancy, based on mid-sagittal, coronal, and axial views of the fetal head. Diagnosis of cleft lip and CLP is usually easier than that of isolated cleft palate on ultrasound [91]. Early diagnosis allows time to plan and organize surgery, enabling it to be performed as soon as possible after birth.

## Conclusions

Early intervention in cleft lip repair may be beneficial for several reasons. First, it provides aesthetic results that are as successful as those achieved with repairs performed at the standard time, that is, at six months. This is thought to result from intervention at a time when tissues are more malleable and at an early stage, halting the progression of the cleft. Additionally, neonatal patients may exhibit some characteristics of fetal scar healing. Furthermore, early closure of the cleft has a knock-on effect on the closure of the alveolar cleft, if present, as well as on the development of alveolar projection. This improves the aesthetic appearance of the face and is more beneficial than using orthodontic appliances during the interim in standard-time repair. More successful results have been seen when neonatal cleft lip repair is carried out using the Tennison technique compared to the Millard technique, which is associated with a much higher rate of complications. As advancements are made in neonatal surgery, anesthetic risk is continually decreasing, with current data showing no difference in risk between operations performed in the neonatal period and those carried out at the standard time. This, of course, assumes that all patients are screened for underlying or undiagnosed conditions that may complicate the operation. Feeding can begin soon after early cleft lip reconstruction through the formation of a functional mouth, allowing neonatal children to thrive from an early age and creating an environment favorable for wound healing. Early repair is also favored for its positive psychosocial impacts; children and young adults are less self-conscious following successful aesthetic repairs, and there is also an improved mother-infant relationship. Taking all factors into consideration, based on the available evidence, neonatal cleft lip repair may be recommended over standard-time repair.
